# Recovery of Petroleum Brine-Contaminated Soil by *Eleocharis* sp. in a Tropical Marshland^*^

**DOI:** 10.21315/tlsr2024.35.2.7

**Published:** 2024-07-31

**Authors:** Verónica Isidra Domínguez-Rodríguez, Francisco J. Guzmán-Osorio, Liliana Hernández-Acosta, Rodolfo Gómez-Cruz, J. Edmundo Rosique-Gil, Randy H. Adams

**Affiliations:** 1 Academic Division of Biological Sciences, Remediation Laboratory, Universidad Juárez Autónoma de Tabasco, Carretera Villahermosa-Cárdenas Km. 0.5 S/N, Villahermosa, Tabasco 86150, México; 2 National Technology of Mexico / ITS of Comalcalco, Carretera Vecinal Comalcalco – Paraíso Km. 2, Ra. Occidente 3ra. Sección Comalcalco, Tabasco C.P. 86651, México

**Keywords:** Hypersaline, Produced Water, Natural Attenuation, Salt Tolerant

## Abstract

Almost all research on natural attenuation and phytoremediation of sites contaminated with briny produced water has been conducted in temperate climates, however, there is a dearth of information on the use of tropical species for this purpose. It is within this context, that we investigated a spontaneously growing hypersaline spikerush from a contaminated site in southeast Mexico, to determine its soil salinity limits, the relationship between soil organic matter and salinity, and for preliminary documentation of floristic succession with *Typha* sp. for phytoremediation o f brine s pills. Soil was sampled (0 cm–20 cm) three times between 2018–2021, focusing on the end of the dry season (most critical period). The species tentatively identified as *Eleocharis mutata* was tolerant to soil hypersalinity (Electrical Conductivity: 125 dS/m) and appeared to generate a cyclic process of succession to recover areas with soil salinity levels higher than it could otherwise tolerate. A salinity gradient was found between the most heavily contaminated part of the site (“kill zone”, > 212 dS/m), the first *Eleocharis* sp. individuals (125 dS/m), slowly advancing through the main spikerush stand, and finally into a cattail stand (< 8.02 dS/m). Similarly, an inverse relationship between Soil Organic Matter content and soil salinity was observed. This is the first time this species has been identified with a brine spill, its salinity limits determined, and investigated for use in phytoremediation of this kind.

**Highlights**

- A halotolerant sprikerush (*Eleocharis* mutata) spontaneously established itself in a marshy area next to a kill zone that had developed due to achronic discharge of briny process water from a petrochemical plant. Itproved to be extremely halotolerant, maintaining vigorous growth even up to 125 dS/m.
- This spikerush appeared to reduce salinity by sequestration in organic material, and was a crucial element in a floristic succession, paving the way for *Typha* sp. colonisation and ultimately, overcoming the negative impacts of brine water discharge.
- *E. mutata* tolerates salts well withing the range of briny process water in the region and may be included in phytoremediation programs for brine spills.

## INTRODUCTION

During petroleum production, wells are sunk deep into oil reservoirs and hydrocarbons are brought to the surface. In many areas, there is sufficient pressure in the formation to do this without pumping, but in some formations, and in older oil fields, the oily mixture may need to be pumped to the surface. This mixture will contain petroleum and salty water from the geological formation, and in some oil fields, natural gas also. These components are separated, first the gas from the liquid and then the salty water from the petroleum. This salty water is known as produced water, as it is generated during the process of producing petroleum, while in Spanish it is called *agua congénita*, because it is co-generated with the petroleum (SEMARNAT 2005; Amakiri *et al*. 2022; Danforth *et al*. 2020). It is usually separated from the petroleum in a dewatering plant by simple physical processes based on different densities of water and oil, and the non-polar characteristic of petroleum hydrocarbons as compared to water. In addition to the salts, and depending on the efficiency of the separation process, it may also contain some hydrocarbons.

Today, in most places in the world, this salty water is treated by several means (oil/water separators, filtration, reverse osmosis, biofiltration and evaporation) depending on its salinity (Amakiri *et al*. 2022; Gamwo *et al*. 2022). Alternatively, it may be re-used in some industrial processes, including the preparation of drilling fluids. In some areas, if law permits, it is disposed of in deep, frequently old, non-producing wells, and this may help repressurise petroleum formations to increase production. In a few areas, where the salinity of the process water is very low, it may be discharged to inland surface water bodies, or if the salinity is high, into the ocean through bottom diffusers, usually at least two kilometres from shore (SEMARNAT 2005). In this context, salinity refers to the amount of Total Dissolved Salts (TDS) in the process water, and there is a general relationship between TDS and the Electrical Conductivity (EC) in the soil solution (OCC 2021). In practical terms, almost all of this salinity is due to sodium chloride in the process water.

In some areas with poor government oversight, especially in the last century, process water was discharged into canals, marshes, or other low-lying areas, usually causing severe salinity (and sometimes hydrocarbon contamination). In many areas this resulted in complete loss of the vegetative soil cover and then severe soil erosion (Lassalle *et al*. 2019). In soils, this salinity is usually determined by preparing a saturated paste of the soil, filtering the soil liquid, and determining the EC in the recovered soil water from the saturated paste. Soils with high EC in the saturated water, will have a high proportion of soluble salts (in brine spills, almost all from sodium chloride).

However, even in areas with reasonable government oversight and proper techniques to minimise the risk to the environment, spills still do occur sometimes, especially from pipeline and valve failures in the infrastructure that transports the process water to deep well injection points. This can result in excess soil salinity (contamination) in the spill zone. In some areas, this may not be so much of a concern if the salinity is relatively low and there is heavy precipitation (especially in humid tropical and subtropical areas), but in others, the degree of impacts may be great and last for many decades, even a century (Dornbusch *et al*. 2020). The range of salinities in process water in the U.S. have been measured between <1000 mg/L Total Dissolved Solids (TDS) to 460,000 mg/L TDS or more (approx. <660 – 303,600+ μS/cm), (OCC 2021; Newell & Connor 2006, Adham *et al*. 2013).

The restoration of these legacy sites (sites contaminated due to poor historical practices), and sites contaminated due to more recent spills, may be achieved through natural attenuation, or phytoremediation if the contamination is not too great and if the Precipitation-Evaporation Index (PEI) is favourable, by leaching out salts. Alternatively, this may be accomplished by chemical amendment (Newell & Connor 2006). In some very severe cases, soil replacement and/or physically producing a capillary break in the soil profile may be necessary.

Typically, the natural attenuation or phytoremediation methods (which are the least expensive) are only recommended for humid sites with a Precipitation-Evaporation Index of more than at least −100 mm/year and with salinities <16 dS/m (Newell & Connor 2006). However, these recommendations have been made for the U.S. climates, typically in the humid/subhumid moisture regime and in the subtropical to temperate temperature regime (Kartesz 2015). However, in the tropical monsoon climate of southeast Mexico, we have observed natural attenuation at a site with much higher salinities.

It is within this context, that we investigated a spontaneously growing hypersaline sedge from SE Mexico, to determine its salt tolerance and capacity to withstand spills from saline produced water from regional sources, as well as performing preliminary research into the relationship between salinity, organic matter production, and floristic succession. In this context this concept is novel –the use of marshy, hypersaline tolerant plants to promote a phyto-restoration solution for low-lying areas contaminated with saline process water in tropical and subtropical environments.

This sedge, the spikerush *Eleocharis mutata (L.)* Roem. & Schult., was identified as being highly salt tolerant and with a capacity to naturally attenuate an area that was previously affected by chronic hypersaline discharge due to historical practices from a petroleum dewatering unit in western Tabasco state, Mexico (Currently, this discharge is discontinued, this is a legacy issue). For the potential use of this species to recover areas impacted by process water, it was considered important to define its salt tolerance and compare this with the salinity of produced water in the region. It was found that there was a direct relationship between salinity reduction in the soil and soil organic matter content, which appeared to be related to phyto-restoration by the spikerush and floristic succession by a cattail species. To our knowledge, this is the first time this limit has been determined for this spikerush species, and the first time it has been identified with areas contaminated with process water from a petroleum industrial complex discharge.

The objectives of this investigation were to:

1. determine the soil salinity limits of the spikerush,
2. the relationship between soil organic matter and soil salinity,
3. develop a salinity gradient based on distance from the “kill zone” (area without vegetation), as well as the type of vegetation, and
4. produce preliminary documentation of floristic succession for phyto-restoration of a brine spill.

## MATERIALS AND METHODS

### Description of the Study Area

**Figure 1:**
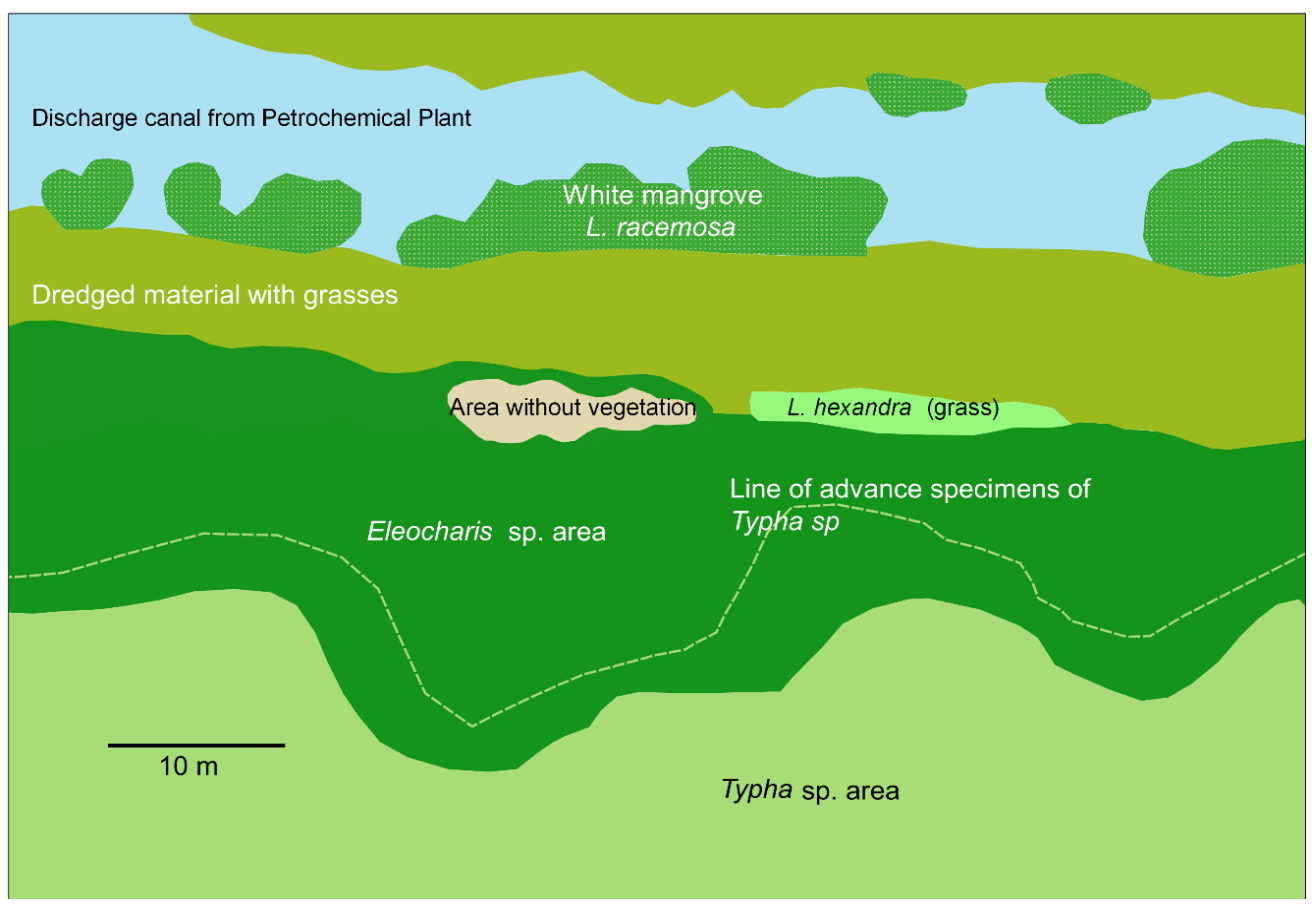
Photomap of the site showing the distribution of the different vegetation types. The top of the figure is North. The discharge point was approx. 320 m East (to the right), of the middle of the area without vegetation.

The study site is adjacent to the La Venta Gas-Processing Complex (*Complejo Procesador de Gas La Venta*), in La Venta, Tabasco, Mexico. Today this site functions mostly to process natural gas, but originally it was developed to process oil and gas from local oil fields. For many years, the produced water from the petroleum dewatering unit was only poorly treated to remove some hydrocarbons, and then discharged into a low-lying marshy area. Historical investigation by García-López *et al*. (2006), indicated that previously this area was a cattail marsh predominated by a *Typha* sp., but following the saline water discharge and canal construction (connecting it to mangrove areas of the Tonalá River), it was transformed into a saline marsh in the upstream area, and a white mangrove forest (*Laguncularia racemosa*) nearer to the river. Some other marsh species were previously found near the site. In previous research conducted adjacent to the study site, about 100 m to the West, we identified a *Fimbristylis* sp. that appeared to be tolerant to hydrocarbon contamination and high alkalinity (Adams *et al*. 2008). This sedge was found to be intermixed with *Typha* sp. and in some areas, with the spiny bush mucal (*Machaerium falciforme* Rudd (Barba *et al*. 2013; GBIF n.d.). None-the-less, closer to the industrial complex, about 100 m to the east, a “kill zone” was found, without vegetation. This area was also found to be covered with a salty crust during some years, during the dry season (Figs. 1, 3 and 4).

### Identification of Resistant Species

**Figure 2:**
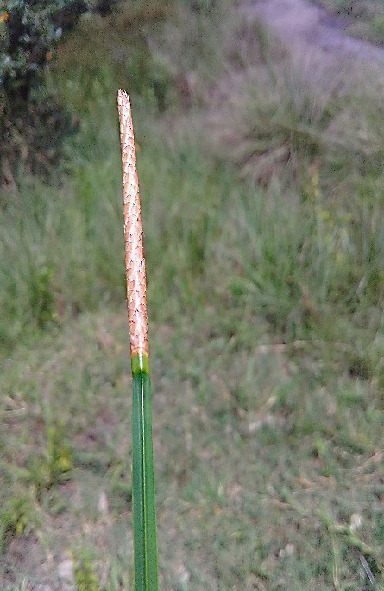
Close-up of an individual of Eleocharis sp. found at the study site.

The selected species has been described in the introduction. It will be described more detail in the results section. The species was tentatively identified using the key presented by Angelo *et al*. (2020), confirming by comparison to photographs from the same source (Fig. 2).

### Research Design

This contaminated site was investigated in an iterative manner, trying to concentrate the research on the most sensitive time of year for soil salinity – at the end of the dry period and before the monsoon rains. This consisted of a preliminary sampling (June 2018) to develop a site photomap defining approximate locations of various kinds of vegetation, and evaluate soil salinity, soil organic matter and soil compaction. Laboratory analysis of the soil samples was conducted to determine soil salinity and percent soil organic matter. With these results, the sampling design for the end of the next dry season was made.

This was followed by a sampling the next year (May 2019), to determine the limits to *Eleocharis* sp. growth in terms of soil salinity. This was accomplished at the end of the dry season, just a few days before the first rains. At this time, a salty crust was observed in the area without vegetation (“kill zone”). This sampling was performed by taking only the uppermost soil (samples from 0 cm–5 cm depth), to observe the highest soil salinity caused by the capillary action in the soil and evaporation at the soil surface.

The following year (2020) it was not possible to sample due to COVID-19 pandemic restrictions.

The next year (May 2021), the pandemic restrictions were reduced, and we were allowed to sample. At this time, a confirmation sampling was made to check the results on limits to *Eleocharis* sp. growth, and also to investigate the relationships between vegetation type and floristic succession (by *Typha* sp.), as well as Soil Organic Matter vs. soil salinity.

### Sampling Periods

**Figure 3:**
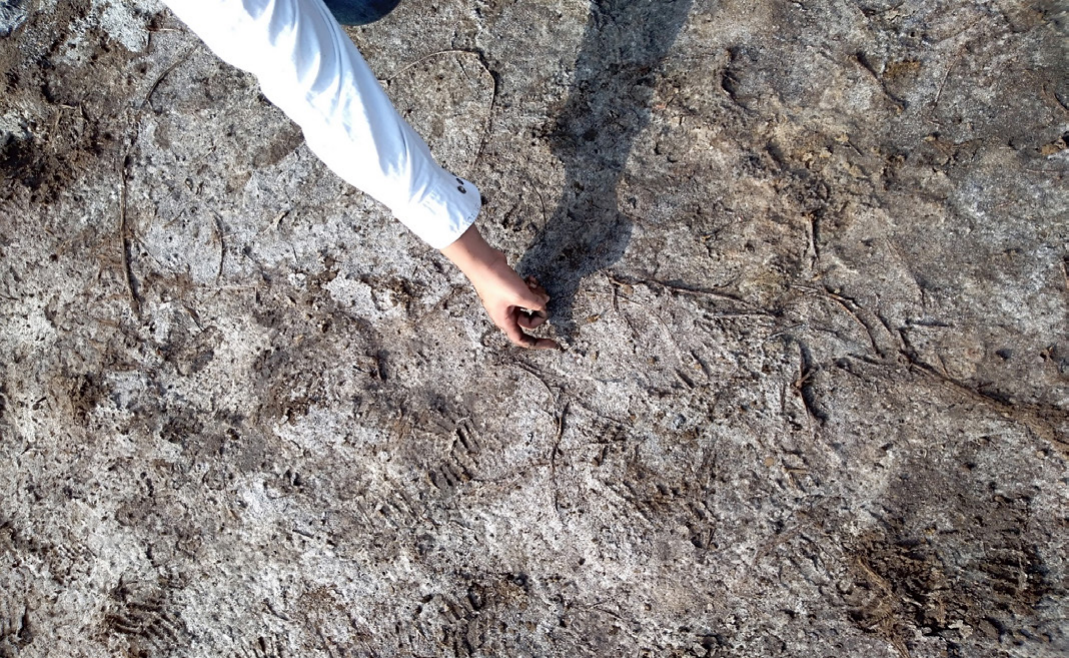
Salty crust observed in kill zone (second sampling period).

Table 1 shows the sampling dates and a description of some of the details observed during sampling. Fig. 1 is a reconstruction of the scenario at the site affected by soil salinity but taking into account features observed in the different samplings, because each sampling had its particularities. In the 2018 sampling, around the area without vegetation, dry *Eleocharis* sp. specimens were found, and this area extended to the west. In 2021 the area shown for *L. hexandra* (more predominant in 2019), was only a line, with the kill zone more open to the east. In the 2019 sampling, several individuals of *Typha* sp. were found to be advancing north of the major *Typha* sp. stand (denoted in Fig. 1 as a dashed line). In the 2021 sampling, the *Typha* sp. had almost advanced completely to this line in a solid stand. Fig. 3 shows sampling conditions from the 2021 sampling.

### Soil Sampling Techniques

#### Zone without vegetation and limit of area with presence of Eleocharis sp

A 10 cm diameter stainless steel split-spoon sampler was used. Samples were collected from the surface to a depth of 20 cm. The samples in the zone without vegetation were collected in the centre of the bare soil, with a separation of approximately 3 m between each sample, in an east-west direction. The samples from the limit of this same kill zone were collected 2 m to the south of samples taken from the centre of the zone in an area that did not show any evidence of plant growth during any time of the year.

**Figure 4:**
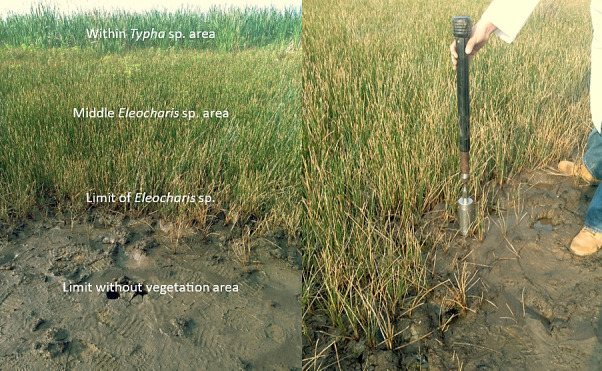
Transition from the zone without vegetation in the foreground to the zone dominated by *Typha* sp. (at left). Sampling at the growth limit of *Eleocharis* sp. (at right). Photographs from 2021.

Samples to determine the limit of soil salinity of *Eleocharis* sp. growth were collected proximally, where the first individuals of the spikerush were encountered, with vigorous growth. In the 2019 sampling, drier conditions were present and vigorous plants were not found until was about one meter into the spikerush stand. In the 2021 sampling, conditions were moister as the rains arrived about three days early. At this sampling (2021), vigorous plants were encountered practically at the limits of the kill zone (Fig. 4), and samples to determine the limit of *Eleocharis* sp. growth were collected only 0.5 m from the no-growth samples. The criterion to define this limit was the presence of healthy, vigorous individuals of *Eleocharis* sp. in the transition zone with and without vegetation.

#### Middle zone of the area with Eleocharis sp. and zone with cattail (Typha sp.) vegetation

A straight shovel was used to make a square cut of 20 cm × 20 cm with a depth of 20 cm, including the roots of the vegetation present. This was necessary due to the peaty nature of the soil in these areas.

Samples in the area with *Eleocharis* sp. were collected in the middle of the vegetation patch, approximately 4 m south of the boundary with the area without vegetation (kill zone); and samples in the area with cattail were collected at a distance between 18 m and 22 m in the same direction, approximately four meters into the cattail stand (the start of which varied).

### Analytical Methods

#### Moisture content

This method is based on the evaporation of water from the sample in a continuous manner, exposing it to a temperature of 100°C. An OHAUS thermobalance, model MB35 HALOGEN, was used to weigh and quantify the moisture percentage. The thermobalance continuously records the weight loss until the sample is at constant weight.

### Electrical conductivity in saturated paste

The saturation extract was obtained using procedure AS-16 established in Mexican norm NOM-021-SEMARNAT-2000 (SEMARNAT 2002), which consisted of adding deionised water to the soil sample up to the saturation point. Subsequently, filtration was performed on Whatman No. 42 paper, using a Buchner funnel and a vacuum line. The electrical conductivity of the recovered extract (saturation extract) was analysed according to method AS-18 of norm NOM-021-SEMARNAT-2000, using a Hanna multiparameter meter, model HI98195.

### Soil organic matter content (% SOM)

SOM was quantified using the calcination method 05.07 (TMECC, 2001). This consisted of eliminating the organic matter from the dry, sieved sample (mesh < 1 mm), and exposing it to a temperature of 550°C for a period of 2 h. Weight differences were determined by performing constant weight of the samples before and after calcination (for periods of 25 min of oven drying at 105°C).

### Field Determinations

#### Soil compaction

Resistance to penetration was determined during the first sampling period using a Dickey John penetrometer according to recommendations of the Penn State College of Agricultural Sciences (Duiker 2002).

## RESULTS

### Site Characteristics and Identification of *Eleocharis* sp

This species was found to be naturally occurring in a wide stand, starting at approximately 250 m from the discharge point of an out-of-use petroleum dewatering unit, in a low-lying, marshy area (18°05’22” N, 94°02’58” W), west of the La Venta Gas Processing Complex (*CPG La Venta*, Tabasco, Mexico).

According to historical studies made by García-López *et al*. (2006), this area was previously dominated by *Typha* sp. before the construction of the petroleum processing complex. This spikerush (*Eleocharis* sp.) had stems with a triangular cross section (triquetrous), and superior seed packets, slightly larger in diameter than the stem, with cartilaginous floral scales. This was differentiated from other genera of Cyperaceae since it had a triquetrous stem (often associated with the genus *Cyperus*), but not the typical inflorescence composed of spikes supported by three peduncles. Furthermore, of the species with the kind of seed packet observed, and with the triangular cross section, there are only two species reported for the region, and only one of these is found in saline environments. It was identified using the key presented by Angelo *et al*. (2020), confirming by comparison to photographs from the same source. In Fig. 2, a close-up is shown of an individual of *Eleocharis* sp. found at the study site.

A similar species has been observed, *E. acutangula* (Roxb.) Shult, which is also found in marshy areas regionally, and has a triquetrous culm. However, the seed back is much thinner, and this species has not been typically associated with hypersaline environments (as has *E. mutata*).

### Preliminary Determinations of Soil Salinity, Soil Organic Matter and Compaction (First Sampling, 2018)

With respect to the parameters measured in the soil, the soil compaction was consistently less with distance from the kill zone, decreasing on average by about two-thirds between the kill zone and the *Eleocharis* sp. stand, and another one-half between the *Eleocharis* sp. stand and the *Typha* sp. area (Table 2). The results of organic matter, electrical conductivity and compaction are all shown in Table 2. Some *Eleocharis* sp. plants were observed in the understory of first several meters within the *Typha* sp. area. Apparently, this area was previously colonised by *Eleocharis* sp. and subsequently by *Typha* sp. There was a power function correlation between %SOM and EC (EC = 766.79*SOM^−0.879^, R^2^ = 0.958), where the soil organic matter content steadily increased as the salinity was reduced. Likewise, this increase in soil organic matter appeared to result in less soil compaction, also showing a power correlation between %SOM and MPa (MPa = 22.649*SOM^−1.432^, R^2^ = 0.994). At any rate, even the most compacted soil did not present sufficient resistance to impede root penetration (Duiker 2002).

It appeared that as the *Eleocharis* species covered the area, it produced a high amount of biomass (noted as having high net primary productivity (NPP) in coastal lagoons in Rio de Janeiro State in Brazil; see Palma-Silva *et al*. 2000). This tends to increase field capacity, sequesters some salt, and paves the way for less tolerant species (*Typha* sp.) (Passago *et al*. 2020, Ding *et al*. 2020, Mao *et al*. 2022). However, when the cattails became established, they outcompeted the shorter *Eleocharis* species for sunlight, and then dominated this area. Also, this species (*Typha* sp.) can have an even greater biomass production and further the salt sequestration (Delattre *et al*. 2022).

### Determination of Salinity Limits of *Eleocharis* sp. (Second Sampling, May 2019)

Non-vegetated soil samples were taken closest to the limits sampling points but in which no evidence for plant growth was observed (in any time of the year). Data for this sampling are shown in Table 3. This sampling date (27 May 2019) was probably the driest and saltiest time of year for these plants, being at the end of the dry season and only a few days before the first monsoon rains. Also, a thin crust of salts was observed on the soil surface in the no-growth area. For this second sampling and definition of limits, the sampling depth was only approx. 5 cm, to determine the concentration of salts due to the capillary affect and evaporation observed at the site (salty surface crust). Also, at this time there was a high variation in the values from within the kill zone, such that the differences are just short of being statistically significant at the 95% confidence interval (*P* = 0.0513), however the tendencies are clear.

This range (up to 121 dS/m) was indeed remarkably high, making this a truly hypersaline-tolerant species. Its high salt tolerance and good capacity to overgrow and colonise areas quickly, makes it an excellent candidate for phyto-assisted restoration of brine impacted sites, especially for marshy areas in tropical and semitropical areas.

### Confirmation of Salinity Limits of *Eleocharis* sp. and Relationship with Vegetation and Soil Organic Material (Third Sampling, May 2021)

At this sampling, no salty crust was evident in the area without vegetation. This year some light rains came about three days earlier than usual. There appeared to be a little advance of *Eleocharis* sp. and green, vigorous plants were found right up to the area without vegetation. Limits samples for *Eleocharis* sp. growth were able to be taken here, in contrast to 2019, in drier conditions, where it was necessary to go into the *Eleocharis* sp. stand about 1m to find green, vigorous plants. Also, the site had not been burned, as in 2019. The distance between the limits to no vegetation and vigorous *Eleocharis* sp. was only about 0.5 m. Additionally, the advance of the *Typha* sp. stand toward the saline kill zone was much greater, as the front of *Typha* sp. had advanced considerably, near to the advance colonisers observed in May 2019.

**Figure 5:**
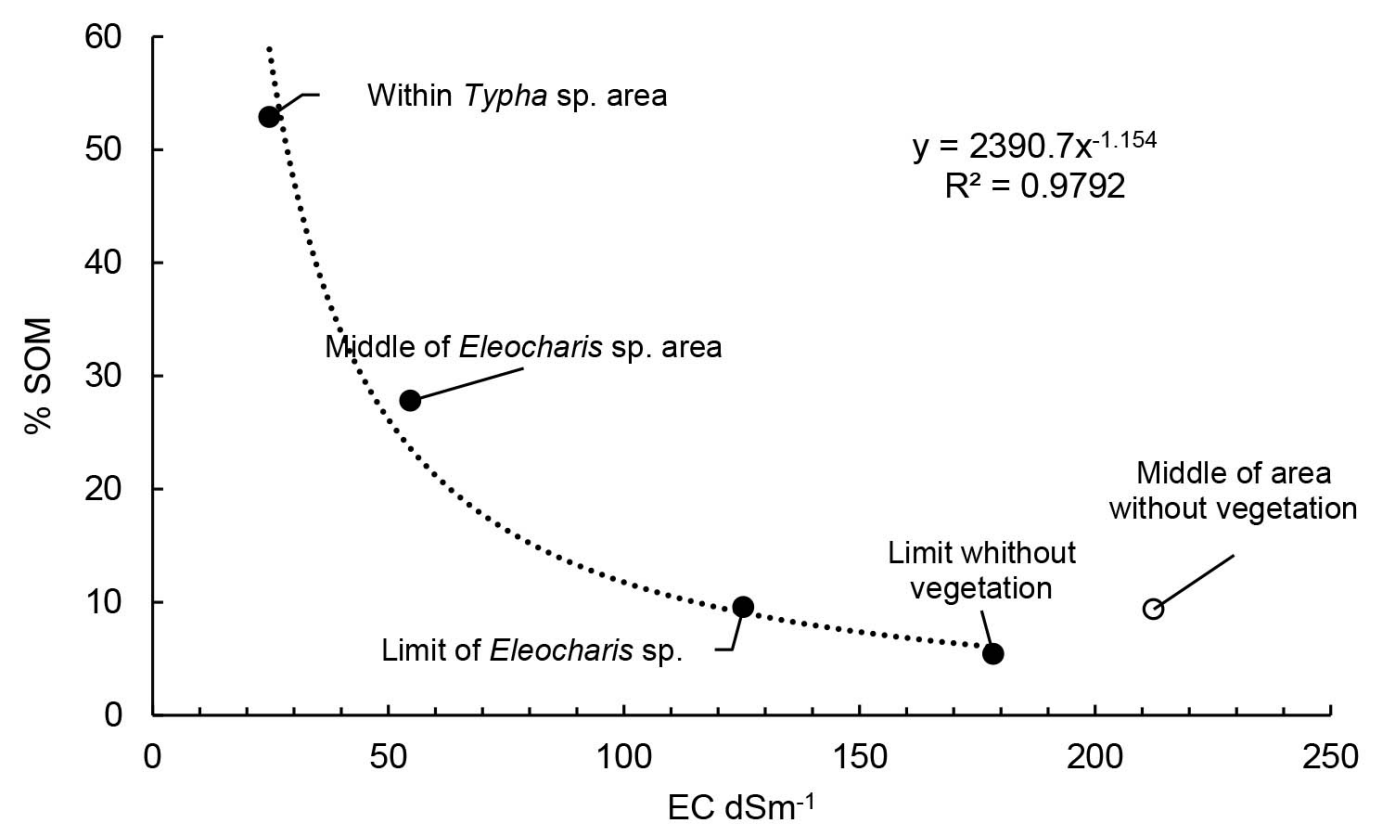
EC behaviour according to the distance from the zone without vegetation to the zone with Typha sp.

The results of parameters of the soil in the third sampling are show in the Table 4, and graphically in Figs. 4 and 5.

The %SOM level presented in the soil of the area with *Typha* sp. was almost two times higher than the level found in the area with *Eleocharis* sp. This is an indicator of the importance of the *Typha* sp. as a secondary succession species that modifies the soil composition in a more significant way.

Fig. 5 shows the conductivity gradient according to the sampling distance from the centre of the kill zone. The absence or presence of each species is also indicated. Of foremost importance is the relatively short distance at which the transition between the area without vegetation and the area with presence of *Eleocharis* sp. occurs (< 1 m), with a drastic change in EC of more than 50 dS/m over this distance.

### Spatial-Temporal Variation of Electrical Conductivity

With respect to the spatial-temporal relationship, the soil salinity is shown in the limit of the area without vegetation, to where *E. mutata* just begins to grow, and in the middle of the spikerush vegetation. The most extreme level of salinity found with *Eleocharis* sp. growth, in the second sampling (121.0 dS/m) was like the average level found at the limit of the vegetation area of this species (125.4 dS/m) in the third sampling. In this context, it is important to emphasise that the most extreme time of the year when soil salinity will affect the vegetation is after the prolonged drought during the spring, in the monsoon climate of the study area. During this season (late March to late May) there is almost no precipitation, extreme temperatures (afternoon highs approx. 35°C–41°C) and high evaporation. The capillary action of the soil wicks salty soil moisture to the surface where it evaporates, leaving salt accumulation (such as the crust observed in 2019). It is probable that the intensity and duration of these processes varies from year to year, according to yearly conditions during this season. This was especially noticeable in the sampling between 2019 to 2021. Even though both samplings occurred in late May, the conditions in 2019 were much more extreme. This was also noted with respect to the distance into the spikerush stand that it was necessary to go to find green, vigorous plants. In 2019, it was necessary to go about one meter into the stand, whereas in 2021 green vigorous plants were found right up to the edge of the kill zone. In any case, the salinity limits found in both dates using this criterion for sample location gave remarkably comparable results for the tolerance salinity, in the range of 121 dS/m–125 dS/m.

## DISCUSSION

In an evaluation if this impacted area, it is important to mention what background levels of soil salinity and soil organic matter content. While this was outside of the scope of the present study, we have determined these values previously in nearby white mangrove forest (*Laguncularia racemosa*), (approx. 250 m to the West, Zavala *et al*. 1999), and in a nearby cattail marsh (approx. 500 m to the South, Zavala *et al*. 1999). In the mangrove forest the soil type was Solonchak (IUSS 2022), with an EC of 52.5 dS/m, and with 17.9 %SOM; while in the cattail marsh the EC was very low (0.2 dS/m) with 82.4 %SOM. This study was conducted during an extreme drought year, and more common levels for mangroves in the area are probably much less. In a black mangrove forest (*Avicennia germinans*) from the north-central part of Tabasco state, a salinity value of 25.9 dS/m was reported (Adams *et al*. 2002). Based on this local literature, it would appear that salinity in mangrove areas ranges about 25 dS/m–30 dS/m. It is noteworthy to mention that these are data from uncontaminated sites in which no relationship was observed between %SOM and salinity. Generally, background %SOM in nearby wetlands ranges from about 18–80% and in soil salinity in mangroves from about 25 dS/m–30 dS/m, but can be almost double in an extreme drought year. Nonetheless, even in such extremes, the soil salinity in local mangroves is less than one-half of that found as the salinity limit for the growth of the *Eleocharis* sp. studied in the present research (125 dS/m).

One factor that could be affecting the successful advance of *E. mutata* at this site (as compared to sites in temperate climates) is the Precipitation-Evaporation Index, in this tropical monsoon climate (Köppen classification system). In the modified Thornthwaite classification system, the site climate is considered perhumid and has a Precipitation-Evaporation Index of about +1210 mm, annually (net). This will certainly reduce water stress caused by the salt, even though during some months there is a deficit. According to Ruíz-Álvarez *et al*. (2012), the site has an annual deficit of about 90 mm, most of which occurs between late March and May.

In Brazil, several reports of *Eleocharis mutata* from coastal areas have been made, one in particular on hypersaline environments (Brito Marques *et al*. 2014). In that report, several other species of the Cyperaceae family were also mentioned including, *Fimbristylis* sp. Vahl and *Eleocharis caribaea* (Rottb. Blake), most commonly known as *E. geniculata* (GBIF n.d.). Both of these later species from hypersaline environments are clearly distinguished from *E. mutata* in that they do not possesses triquetrous culms. In Brazil, this species has also been known for recolonising perturbed areas quickly, especially considering its relatively high net primary productivity (NPP) even in saline environments (Palma-Silva *et al*. 2000).

Natural stands of this *Eleocharis* species from saline areas nearby (near La Venta, Tabasco), have also been reported in uncontaminated areas near mangroves along the Tonalá River (personal communication of D. Rosen mentioned in Angelo *et al*. 2020), also in Sánchez-Cordero *et al*. (2020) and GBIF (n.d). This species has been reported mostly in the Caribbean, southern Mexico, Central America, north and central South America, in some countries in Western Africa (Angelo *et al*. 2020; GBIF n.d.), as well as south Florida (U.S.) and in SE Texas (U.S.) (Rosen & Jones 2004).

If, as suggested, this species may be used to treat brine-spill contaminated sites, it is important to compare its salinity tolerance to the salinity of process water locally. It is difficult to know exactly what range of salinity might be found in the soil shortly after a brine spill, but some estimate could come from data on the process water itself. In our work in Tabasco, we have found EC in process water in the range of 104.2–130.0 dS/m in the Dos Bocas Marine Terminal, TMDB (unpublished report – Tecnologías Ambientales Alemanas/BAUER Resources GmbH 2015). The range of some of the most pertinent values in the process water is shown in Table 5.

**Figure 7:**
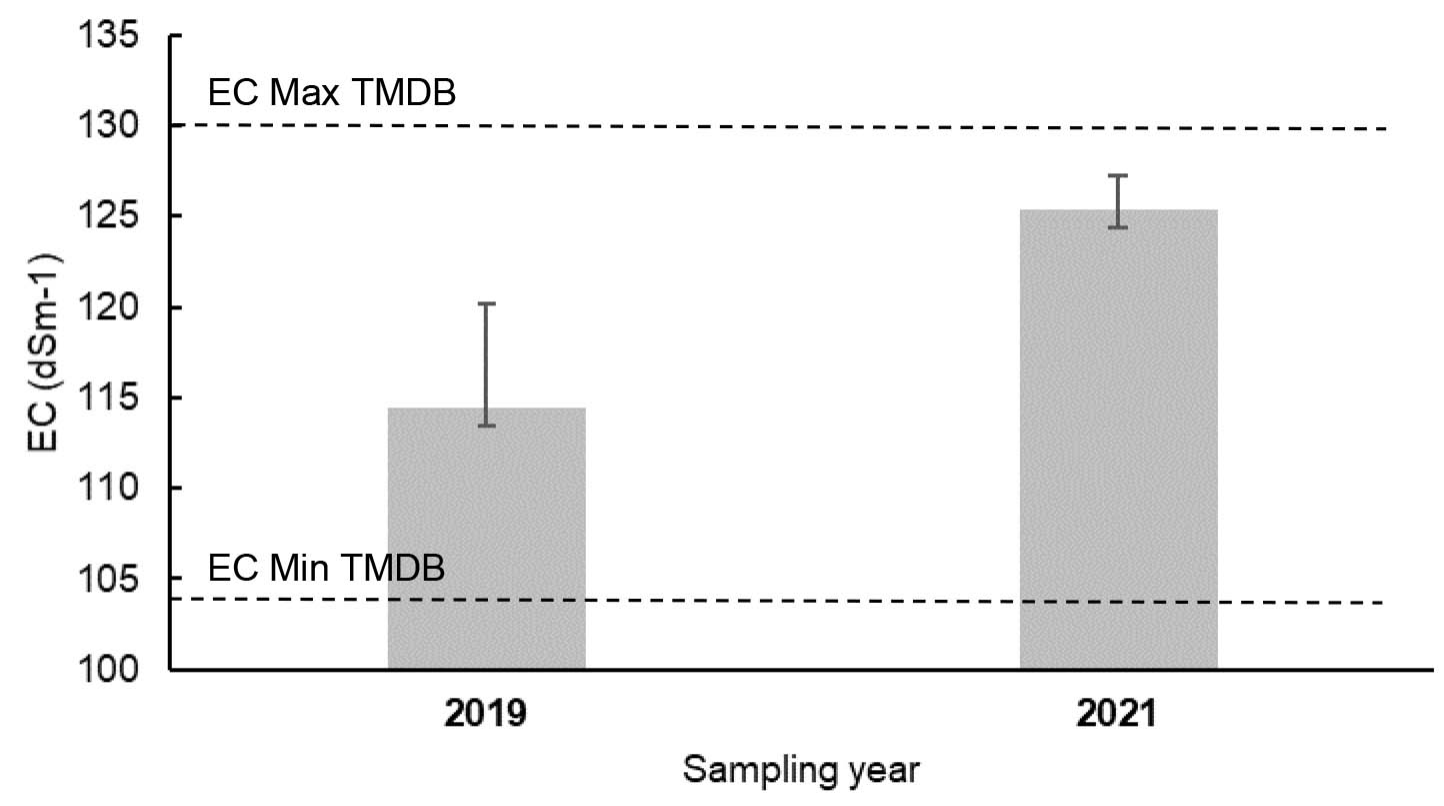
Comparison of tolerance levels of Eleocharis sp. with those of TMDB process water.

In Fig. 7, the range of salinity in process water from TMDB is compared with of tolerance levels of *Eleocharis* sp. in the present study. As seen in this figure, for many brine spills in marshy areas, at least locally, this species could be used to establish a vegetative cover very soon, if not immediately after the spill. This could be useful to avoid soil erosion, and then allow natural attenuation to take its course and slowly reduce soil salinity, establishing a vigorous plant growth. Combined strategies incorporating some organic peaty into the soil prior to planting, may also prove useful. It is important to note that this application is being considered to treat soil contaminated by brine spills or inactive effluents from legacy sites, not to treat the process water in active effluents. Frequently, at least in the tropical monsoon climate of the study area, the soil salinity shortly after a brine spill is much less than the conductivity of the brine spilled, often in the range of 20 dS/m–80 dS/m in the soil. At the study site in La Venta, it was much higher since this was contaminated not due to a spill, but an effluent discharge over several decades, which was exacerbated due to local topography (concave, low-lying area and evaporation).

It is probable that the major interaction of the vegetation is dominated by the soil salinity which is markedly higher at the end of the drought. In the first sampling (after of some rains), unlike the end of the drought period, the conductivity was lower than what this plant can tolerate, but is possible that the sedge was not present in the without vegetation area because it requires a repopulation process that we were not able to observe, due to the short time elapsed between the end of the dry season and the sampling date (first weeks of the rainy season).

**Figure 8:**
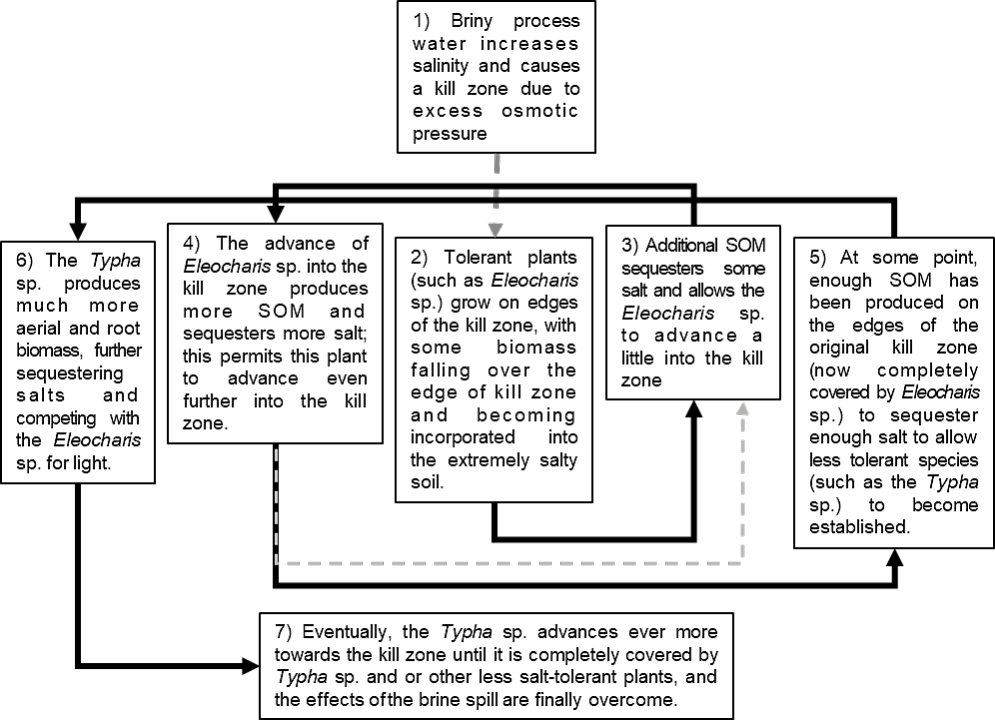
Conceptual diagram of the cyclic process of species interaction for salt spill recovery.

**Figure 9:**
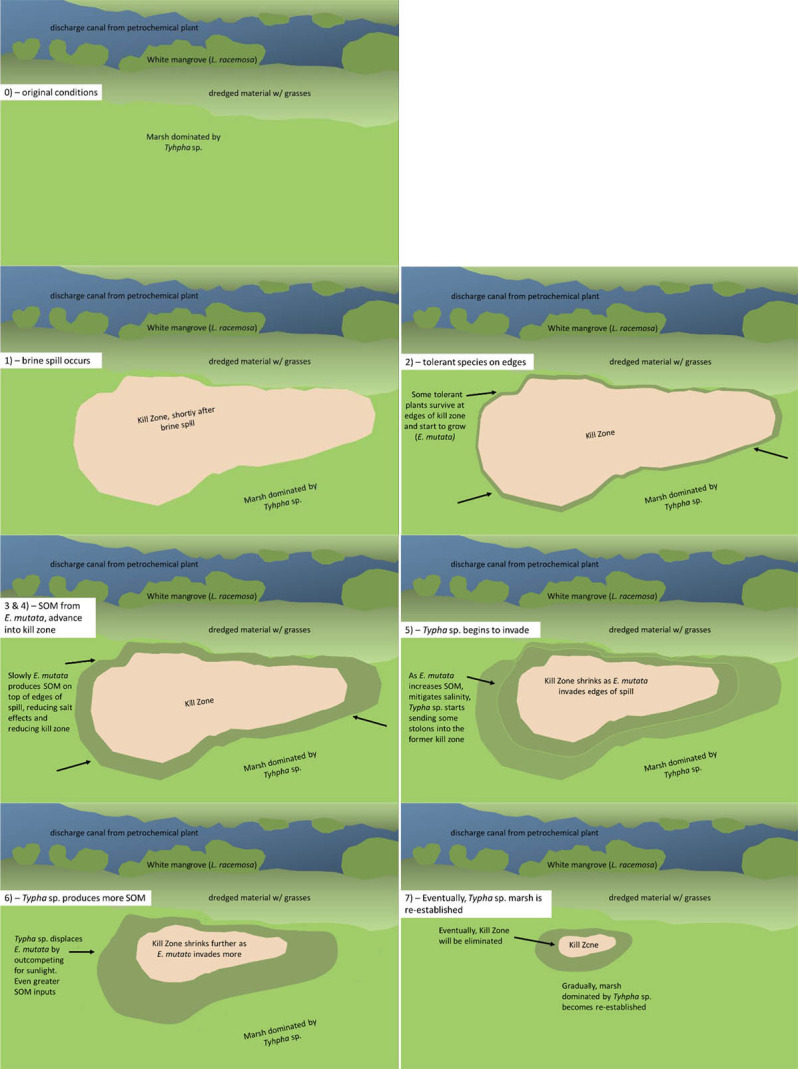
Representation of natural attenuation of a brine spill by E. mutata and Typha sp.

In this study there was a reverse relationship between the soil salinity and soil organic matter content. During the recovery of brine spills, the effect between EC and %SOM may be cyclical. Fig. 8 shows a diagram to explain the proposed cyclical process. This is also explained in a series of hypothetical maps in Fig. 9. Immediately after a brine spill, the local vegetation is killed off, due to excess osmotic pressure and water stress. At the edges of the spill, some more resistant plants may survive (such as *E. mutata*). As these plants grow, some of the aerial biomass might fall into the kill zone and may increase the %SOM in the soil. Likewise, some of this biomass will fall around the plant itself, and some increase in %SOM may also result from transformation of root exudates into humic substances by rhizosphere microorganisms. It has been well established that an increase in %SOM may sequester some salts (Passago *et al*. 2020; Ding *et al*. 2020; Mao *et al*. 2022) and allow the plants to better establish themselves. A subsequent decrease in soil salinity will allow plants to gain ground in the unvegetated area, repeating the cycle. When enough SOM has been placed into the soil by the most tolerant species (like *E. mutata*), and the soil salinity is mitigated, other less tolerant plants may then invade the area (like the *Typha* sp.), and displace the original colonisers of the brine spill, usually by outcompeting them for sunlight. As is often the case, these taller, larger plants have even greater photosynthetic capacity, and can produce even more SOM. This may continue until the secondary species completely recovers the area of the brine spill, which is then, effectively overcome. In the long term, other vegetative species may also invade the area, increasing the biodiversity.

Considering that *E. mutata* is typically associated with mangroves, but that its global distribution is also a little more toward temperate areas (including SE Texas, for example, Rosen & Jones 2004), there are several petroleum producing areas globally where *E. mutata* could potentially be used to mitigate spills of process water from petroleum production and refining. These could include coastal areas, as well as inland areas that may have moist, marshy conditions, such as:

1. The Gulf of Mexico coast from Campeche to southern Tamaulipas, and in SE Texas to Louisiana.
2. Coastal areas in NE Brazil and in Venezuela.
3. Some inland areas of Venezuela, Colombia, Ecuador, Peru and Brazil in the Llanos region and western Amazonia, as well as the Argentinian Mesopotamia.
4. Sumatra, Borneo, Papua and northern areas of Australia.
5. West Africa, including Nigeria, Angola, Gabon; and also the Sudd region of South Sudan in eastern Africa.
6. Gujarat and Assam states in India.
7. The Persian Gulf, possibly as far north as Kuwait.
8. Some coastal areas of southern China, especially in Guangdong, Hainan and Taiwan.

When comparing the results of the present study to past work, two lines of research are pertinent: (1) phytoremediation of brine spills, and (2) studies on extreme halophiles. With respect to the first, there is very little recent research, and the most comprehensive compilation was prepared for the American Petroleum Institute for the treatment of brine spills in the U.S. (Newell & Connor 2006). In this guide, plants are recommended basically for areas in the humid/subhumid moisture regime and in the subtropical to temperate temperature regime (see Table 6). Furthermore, natural attenuation or phytoremediation is not recommended for sites with greater than 16 dSm^−1^ of soil salinity. More recently, preliminary work was undertaken to find species with the potential to recover brine spill areas in Saskatchewan, Canada (Gerrard *et al*. 2005), in the northern prairies. One of the species is of the genera *Suaeda*, which is known to have many halophiles, however this species (*S. calceoliformis*) is only known to inhabit temperate areas. None-the-less, the other species identified in this study (*Distichlis stricta*), is known to grow in both temperate and tropical environments (CONABIO 2009). Unfortunately, the salt tolerance of this species was not mentioned in the paper. Another more recent study (Jesus *et al*. 2015), reports on the capacity of a wide variety of species that may be used to recover salt-affected soils. However, this review was not oriented to brine spills but soil affected by other natural, and man-made causes, and none of the species mentioned in the paper tolerated more than 19 dS/m.

With respect to the literature on halophytes, one pertinent study was made on an uncontaminated area nearby the study area within a white mangrove forest (*Laguncularia racemosa*, Zavala *et al*. 1999). What stands out about this study is that it was made during an extreme drought caused by the El Niño effect, locally in 1998. During this event, the soil in mangrove forests was not saturated and soil water was not found until approximately 90 cm into the soil, making this truly an extreme year. Nonetheless, this species tolerated more than 50 dS/m, more than double that mentioned for the species in the literature on phytoremediation. En even more extreme halophile has been discovered recently (Palchetti *et al*. 2021) in salt pans in the high Andes, in South America. This species grows at 2,000 m–4,000 m above sea level, and in arid conditions (< 50 mm of precipitation annually). This halophile, *Lycium humile*, known in the local indigenous language as Walcha, was shown to tolerate pulses of up to 1,000 mM NaCl in the laboratory, approximately equal to 89 dS/m. In comparison, in the present study, we observed under field conditions this spikerush (*E. mutata*) repeatedly tolerating about 40% more salinity (about 125 dS/m) than the extreme halophile found in the Andes, at least at some time during the year. This was observed at the same time of year, in two successive years.

This may be among the most salt tolerant species of halophytes ever encountered and merits more research. Among plants adapted to naturally saline environments, the maximum tolerance encountered appears to be about 90 dS/m in the soil extract, (approx. 1,000 mM NaCl in pore water). It would appear that there are very few naturally occurring habitats that exceed this level. That we encountered a naturally growing halophyte that tolerates such extreme salinity appears to be entirely fortuitous and due to the natural selection pressure on nearby vegetation, caused by a completely anthropomorphic source (decades long discharge of process water, low-lying, concave landscape, and salt accumulation through evaporation). However, even though the site is extremely saline, it is very moist almost all year long, which may also play a factor.

## CONCLUSION

The *Eleocharis* sp. studied, tentatively identified as *E. mutata*, is truly a hypersaline plant (soil salinity up to 125 dS/m). At this site, the salinity and SOM were inversely proportional and closely related to vegetation type. Although *E. mutata* was the first colonizer, the *Typha* sp. also plays a vital role in site recovery as a secondary successional species.

At this low-lying site in the tropics, phyto-restoration naturally occurred with the *E. mutata/Typha* sp. association, even at soil salinities much higher than that reported in the literature using temperate species and has a high potential for the recovery of soils affected by briny process water spills under marshy conditions in the tropics. However, it is important to conduct systematic studies in the future to confirm the interaction between plant and soil, to determine the most crucial factors in the succession process.

## Figures and Tables

**Figure 6:**
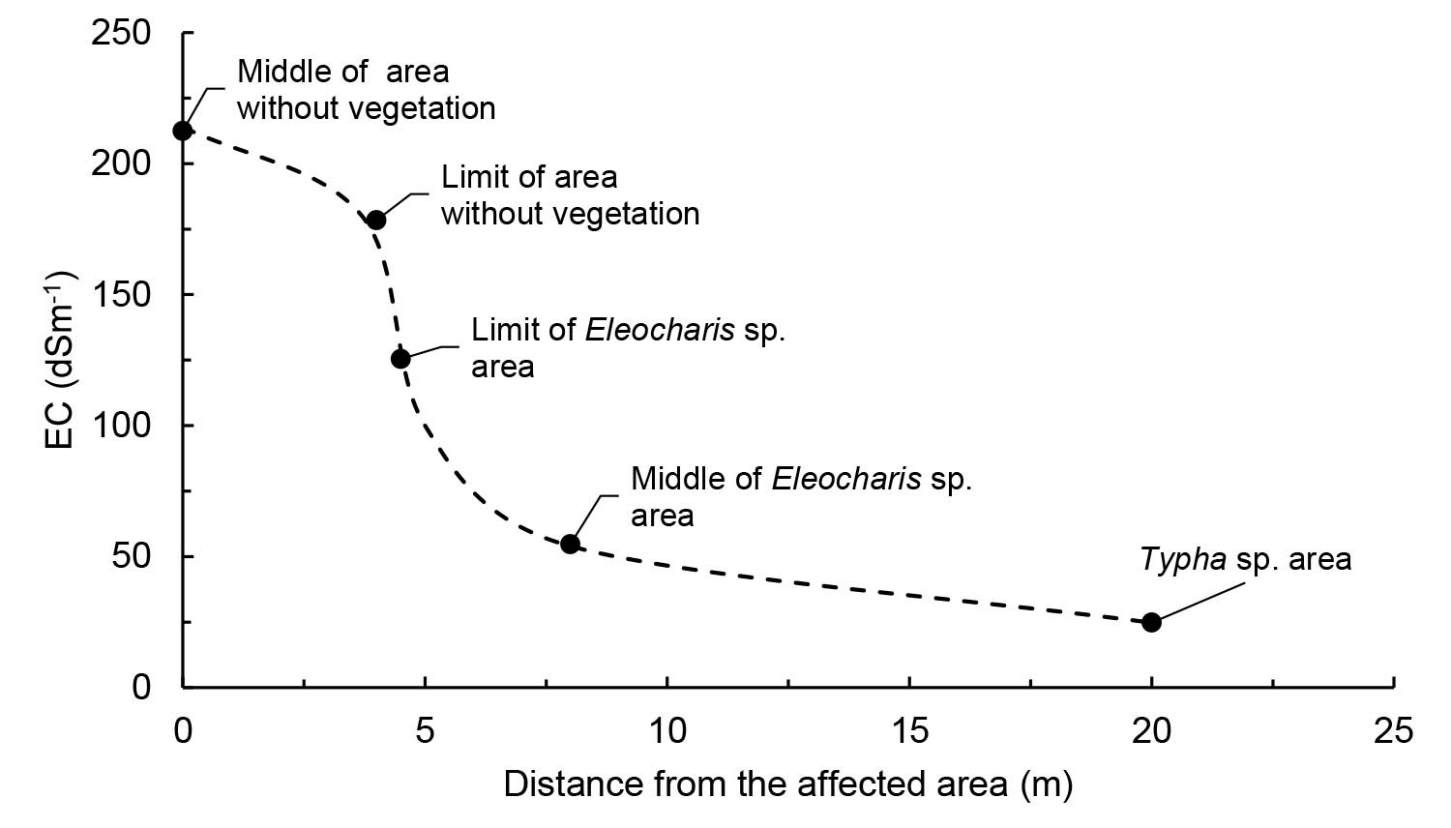
Conductivity gradient according to the sampling distance.

**Table 1:** Sampling dates and a description of some of the details observed during sampling.

Sampling	Weather characteristics	Sampling details
First sampling 15 June 2018	Sampling period after some rains (end of May, early June).	Three samples were taken in each area and determinations on SOM and EC (in the saturated paste) were made. Also, resistance to penetration (John-Dickey penetrometer) was determined. Using GPS, field observations and Google Earth Satellite images, a photomap was developed of the area including the canal, levee, mangroves, no vegetation zone, dry and green *Eleocharis* sp. and *Typha* sp. stands, as well as sampling points.
Second sampling 27 May 2019	End of dry season and 4 days before first rains. This is probably the driest and saltiest time of year for these plants.	Only sampled area without vegetation and limits to growth. Three samples were taken in each area. Limits were determined as green, vigorous plants closest to the kill zone. Apparently, ranchers had burned the site a few weeks earlier.
Third sampling 21 May 2021	Sampling just after first rains (about two days of light, occasional showers, which came early this year). Site much moister than in previous sampling two years earlier.	Samples were taken in the middle of the area without vegetation, about 4 m into the *Eleocharis* sp. stand, and 12 m, 14 m and 16 m more from there south, well into the *Typha* sp. stand, to be in the approximate location of the sampling from 15 June 2018.

**Table 2:** Determination of % SOM, soil salinity, and compaction of the soil in the preliminary sampling. Values are averages of three replicates with standard deviations.

Area	EC-sat. paste (dS/m)	%SOM	Resistance to Penetration, Average 0 cm–38 cm (MPa)
Saline area w/o vegetation	91.0 ± 14.0	12.6 ± 1.4	0.64 ± 0.16
Middle of *Eleocharis* sp. area	37.5 ± 3.8	25.0 ± 5.0	0.20 ± 0.02
Within *Typha* sp. area	27.3 + 5.6	49.6 + 9.7	0.09 ± 0.02
*P* =	0.0273*	0.0009*	0.0257*

**Table 3:** Determination of soil salinity limits to Eleocharis sp. growth (second sampling),

Area	EC (dS/m)	EC range (dS/m)
Limits without vegetation	145.90 ± 58.17	92.6 – 207.95
Limits of *Eleocharis* sp. Growth	114.48 ± 5.75	110.1 – 121.0
*P* =	0.0513	

**Table 4:** Confirmation of limits to Eleocharis sp. growth and relationship to vegetation and %SOM (third sampling).

Area	EC(dS/m)	%SOM
Middle of area without vegetation	212.45 ± 15.48	9.37 ± 4.07
Limit without vegetation	178.40 ± 10.55	5.41 ± 1.08
Limit of *Eleocharis* sp.	125.40 ± 1.83	9.55 ± 4.53
Middle of *Eleocharis* sp. Area	54.72 ± 2.55	27.79 ± 7.48
*Typha* sp. Area	24.77 ± 3.45	52.87 ± 11.75
*P* =	0.0317*	0.0361*

**Table 5:** Range of values for salinity, pH, Oil and Grease, and some heavy metals in process water from the Dos Bocas Marine Terminal (TMDB).

Parameter	Minimum	Maximum
EC (μS/cm)	104,166	130,000
Total Suspended Solids (TSS, mg/L)	60	440
Total Dissolved Solids (TDS, mg/L)	96,000	120,000
pH	6.70	8.04
Oil and Grease (O&G, mg/L)	5	119
Cr (total, mg/L)	0.04	0.09
Cd (mg/L)	Not detected (< 0.004)	Not detected (< 0.004)
Pb	Not detected (< 0.1)	Not detected (< 0.1)

*Source*: Tecnologías Ambientales Alemanas/BAUER Resources GmbH 2015

**Table 6:** Comparison of species recommended for phytoremediation of brine spills and extreme halophytes.

Reference	Species	Climate	Salinity
Newell & Connor (2006) (American Petroleum Institute); Phytoremediation of brine spills.	Alkali Sacaton *Sporobolus airoides*	Temperate	< 16 dS/m (recommendation)
	Basin Wildrye *Leymus cinereus*	Temperate	
	Western Wheatgrass Pascopyrum smithii	Temperate	
	Beardless Wildrye *Leymus triticoides*	Temperate	
	Tall Wheatgrass *Thinopyrum ponticum*	Temperate	
Gerrard *et al*. (2005) (University of Saskatchewan); Screening study for brine spills.	Sea Blite *Suaeda calceoliformis*	Temperate	Tolerance to high Na+ concentrations
	Salt Grass *Distichlis strict*a	Temperate and Tropical	Tolerance to high Na+ concentrations
Jesus *et al*. (2015); Review paper on salt-affected soils (not oriented to brine spills).	Variety of species	Temperate and Tropical	< 19 dS/m
Zavala *et al*. (1999); Ccharacterisation of local soils.	White Mangrove *Laguncularia racemosa*	Tropical	52 dS/m
Palchetti *et al*. (2021); Sstudy on extreme halophiles.	*Walcha Lycium humile*	Extratropical (tropical highlands)	~89 dS/m
This study; Natural attenuation of a brine spill.	Spikerush *Eleocharis mutata*	Tropical	125 dS/m
